# Pro-arrhythmic Effects of Hydrogen Sulfide in Healthy and Ischemic Cardiac Tissues: Insight From a Simulation Study

**DOI:** 10.3389/fphys.2019.01482

**Published:** 2019-12-13

**Authors:** Shugang Zhang, Shanzhuo Zhang, Xiaoshuai Fan, Wei Wang, Zhen Li, Dongning Jia, Zhiqiang Wei, Henggui Zhang

**Affiliations:** ^1^Department of Computer Science and Technology, Ocean University of China, Qingdao, China; ^2^Qingdao National Laboratory for Marine Science and Technology, Qingdao, China; ^3^School of Computer Science and Technology, Harbin Institute of Technology, Harbin, China; ^4^Biological Physics Group, Department of Physics and Astronomy, The University of Manchester, Manchester, United Kingdom; ^5^School of Computer Science and Technology, Harbin Institute of Technology, Shenzhen, China; ^6^Key Laboratory of Medical Electrophysiology of Ministry of Education and Medical Electrophysiological Key Laboratory of Sichuan Province, Institute of Cardiovascular Research, Southwest Medical University, Luzhou, China

**Keywords:** arrhythmia, air pollution, hydrogen sulfide, ischemia, simulation

## Abstract

Hydrogen sulfide (H_2_S), an ambient air pollutant, has been reported to increase cardiac events in patients with cardiovascular diseases, but the underlying mechanisms remain not elucidated. This study investigated the pro-arrhythmic effects of H_2_S in healthy and ischemic conditions. Experimental data of H_2_S effects on ionic channels (including the L-type Ca^2+^ channel and ATP-sensitive K^+^ channel) were incorporated into a virtual heart model to evaluate their integral action on cardiac arrhythmogenesis. It was shown that H_2_S depressed cellular excitability, abbreviated action potential duration, and augmented tissue’s transmural dispersion of repolarization, resulting in an increase in tissue susceptibility to initiation and maintenance of reentry. The observed effects of H_2_S on cardiac excitation are more remarkable in the ischemic condition than in the healthy condition. This study provides mechanistic insights into the pro-arrhythmic effects of air pollution (H_2_S), especially in the case with extant ischemic conditions.

## Introduction

Pro-arrhythmia risks of ambient air pollution have been long established by epidemiological evidences. Inflammation, autonomic nervous system (ANS) changes, and pulmonary or systemic oxidative stress are currently recognized as potential factors responsible for arrhythmogenesis of air pollution (Link and Dockery, 2010; Martinelli et al., 2013); however, the specific effect of air pollution on cardiac functions remains incompletely elucidated owing to the complicated composition of air pollutants (Schlesinger, 2007; Mills et al., 2009; Fiordelisi et al., 2017). Hydrogen sulfide (H_2_S) is a common air pollutant characterized by an odor of rotten eggs and is largely produced in industrial activities, such as food processing, coke ovens, and petroleum refineries. Although epidemiological research has shown association between ambient H_2_S and cardiovascular disease hospitalization and mortality (Godleski et al., 2000; Bates et al., 2002; Finnbjornsdottir et al., 2015, 2016), how it affects the functions of the cardiovascular system is unclear.

Recent studies at the cellular level suggested that NaHS (H_2_S donor) can significantly shorten the action potential duration (APD) of cardiomyocytes, possibly by affecting the ATP-sensitive K^+^ channel (K-ATP) (Bian et al., 2006; Johansen et al., 2006; Zhang et al., 2007; Abramochkin et al., 2009; Chan et al., 2018) and the L-type Ca^2+^ channel (Ca-L) (Sun et al., 2008; Zhang et al., 2012). Experiments on rats have characterized the dose-dependent effects of NaHS on ionic currents of the two channels, *I*_*CaL*_ and *I*_*K,ATP*_ (Sun et al., 2008; Zhong et al., 2010), and suggested that low doses of NaHS (i.e., 100 and 150 μmol/L) abbreviated APD by either an enhanced *I*_*K,ATP*_ (Zhong et al., 2010) or a reduced *I*_*CaL*_ (Sun et al., 2008). Although it is possible that other channel currents may be involved in their experiments owing to the use of a high concentration of intracellular ATP or channel blockers, it is unclear if the observed increase in *I*_*K,ATP*_ or decrease in *I*_*CaL*_ is sufficient to account for the abbreviation of the APD by NaHS.

It is unclear either how NaHS affects the conduction of action potential (AP) through the transmural ventricular wall. It is known that regional difference of cellular property exists in transmural ventricle cells, and such regional difference in cellular property may be altered by NaHS by modulating the intrinsic transmural dispersion of APD and the effective refractory period (ERP) of cardiac tissues, leading to increased tissue susceptibility to arrhythmogenesis. In addition, in ischemic condition, cardiac electrophysiology is remodeled (Shaw and Rudy, 1997; Trénor et al., 2007; De Diego et al., 2008). It is unclear how NaHS affects the conduction of electrical excitation waves in ischemic tissues and modulates the transmural ERP dispersion, leading to an increased pro-arrhythmic effect.

This study aimed to use a mathematical model of the heart (virtual heart) to evaluate the functional impact of H_2_S/NaHS-induced changes in *I*_*CaL*_ and *I*_*K,ATP*_ on the electrical properties of rat ventricular myocardium. Specifically, we modified the Pandit et al. (2001) model of healthy rat ventricular myocytes by incorporating *I*_*K,ATP*_ and actions of NaHS on *I*_*CaL*_ and *I*_*K,ATP*_. Using the model, we simulated the effect of NaHS on cardiac excitation in both control and ischemic conditions with considerations of ischemia-induced alterations to cellular and tissue properties based on available experimental data. Using cellular models, effects of NaHS on cellular AP, excitability, APD, ERP, and transmural dispersion of ERP (ΔERP) were computed. Using one-dimensional (1D) model of a transmural ventricular tissue strand, effects of NaHS on ventricular conduction and susceptibility to arrhythmogenesis were also investigated. Finally, effects of NaHS on the genesis and maintenance of reentrant arrhythmias in a two-dimensional (2D) transmural ventricular sheet model were quantified.

## Results

### Effects of NaHS on Action Potentials via Changes in Individual Ion Channels

**FIGURE 1:**
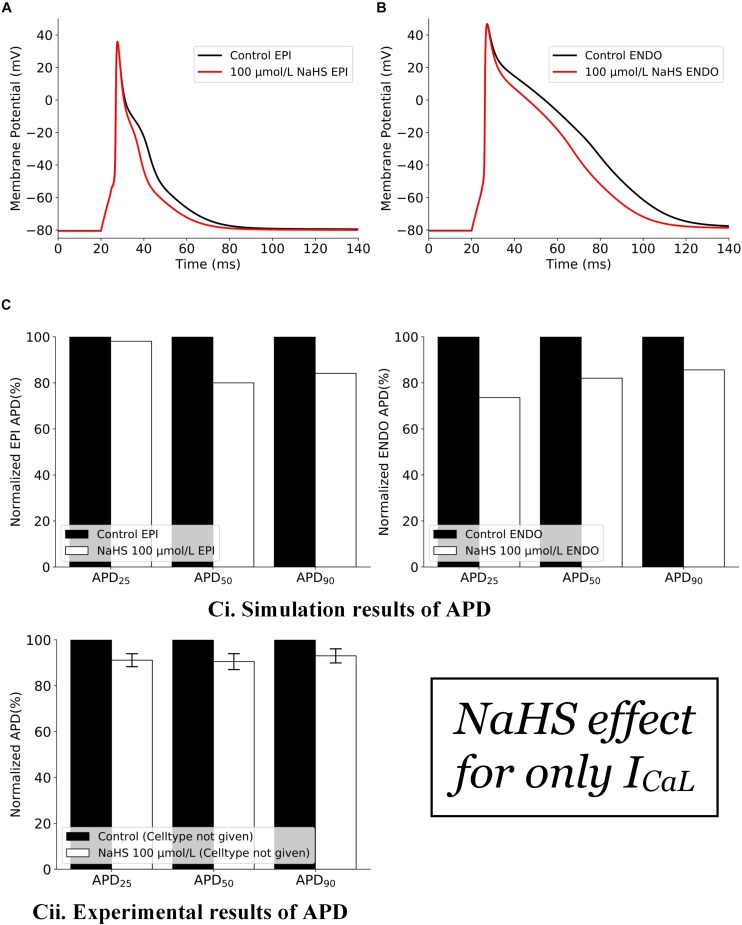
Effects of 100 μmol/L NaHS on AP by its inhibition action on Ca-L channels alone. **(A)** APs of epicardial myocytes in control and NaHS conditions. **(B)** APs of endocardial myocytes in control and NaHS conditions. **(C)** Comparison of APD abbreviation between simulation and experimental data. **(i)** Simulation results; **(ii)** experimental results from Sun et al. (2008) without specifying cell types. AP, action potential; APD, action potential duration.

The effects of NaHS by its action on *I*_*CaL*_ alone are shown in Figure 1. In simulations, the inhibition action of 100 μmol/L NaHS on *I*_*CaL*_ was simulated by Eq. 3, whereas the *I*_*K,ATP*_ was simulated based on Eq. 2 with the a ratio of intracellular ATP:ADP being kept normoxic as in Sun et al. (2008) (see section “Materials and Methods”). It was shown that a decreased *I*_*CaL*_ by NaHS abbreviated the APD for both endocardial (Figure 1A) and epicardial (Figure 1B) cells, with no marked effect on their amplitude or the resting potential. Such simulated changes qualitatively matched with experimental data of Sun et al. (2008), although there are some quantitative discrepancies between simulation and experimental data (Figure 1C), possibly owing to a greater *I*_*CaL*_ current density in the Pandit model (around −11 pA/pF) as compared with the experimental data (only −3.21 ± 0.13 pA/pF), which produced a greater APD abbreviation than the experimental data of Sun et al. (2008).

**FIGURE 2:**
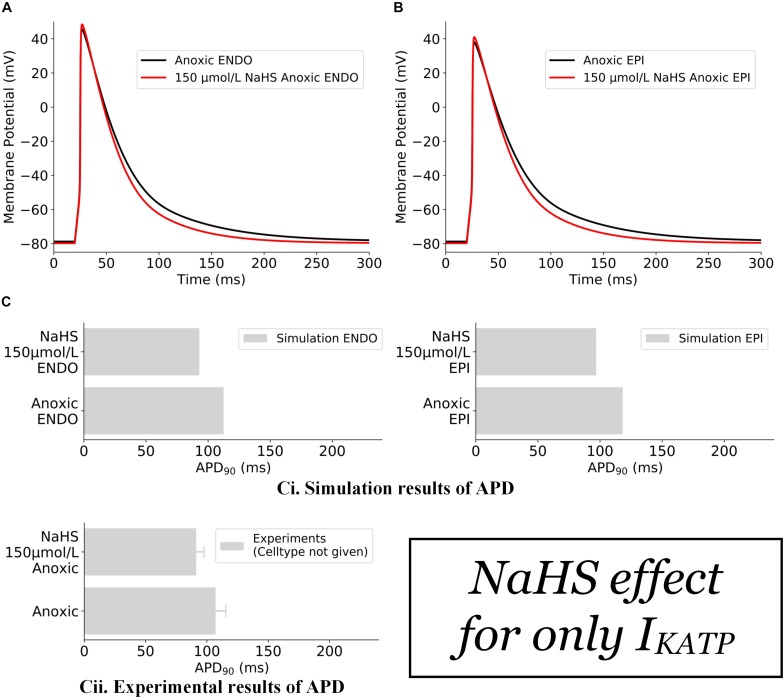
Effects of 150 μmol/L NaHS on AP of anoxic ventricular myocytes via *I*_*K,ATP*_ alone. **(A)** APs of endocardial myocytes in control and NaHS condition. **(B)** APs of epicardial myocytes in control and NaHS conditions. **(C)** Comparison of simulated APD abbreviation with experimental data. **(i)** Simulation results; **(ii)** experimental results from Zhong et al. (2010) without specifying cell types. In the simulations, *I*_*to*_, *I*_*CaL*_, and *I*_*K*__1_ were blocked in accordance with experimental settings. AP, action potential; APD, action potential duration.

The effects of NaHS on *I*_*K,ATP*_ alone are illustrated in Figure 2. In this case, the action of NaHS on *I*_*K,ATP*_ was calculated from Eq. 2 (see section “Materials and Methods”) with a ratio of intracellular ATP:ADP as 200:4.5 for anoxic myocytes to mimic the low ATP experimental setting. It was shown that the simulated hypoxic condition abbreviated APD in both the endocardial (Figure 2A) and epicardial (Figure 2B) cells with the ATP:ADP setting as stated above. The simulated APD abbreviation of both cell types was consistent with experimental data of Zhong et al. (2010) (Figure 2Cii), although no specific cell type was identified in their experimental study.

### Effects of NaHS on APs

**FIGURE 3:**
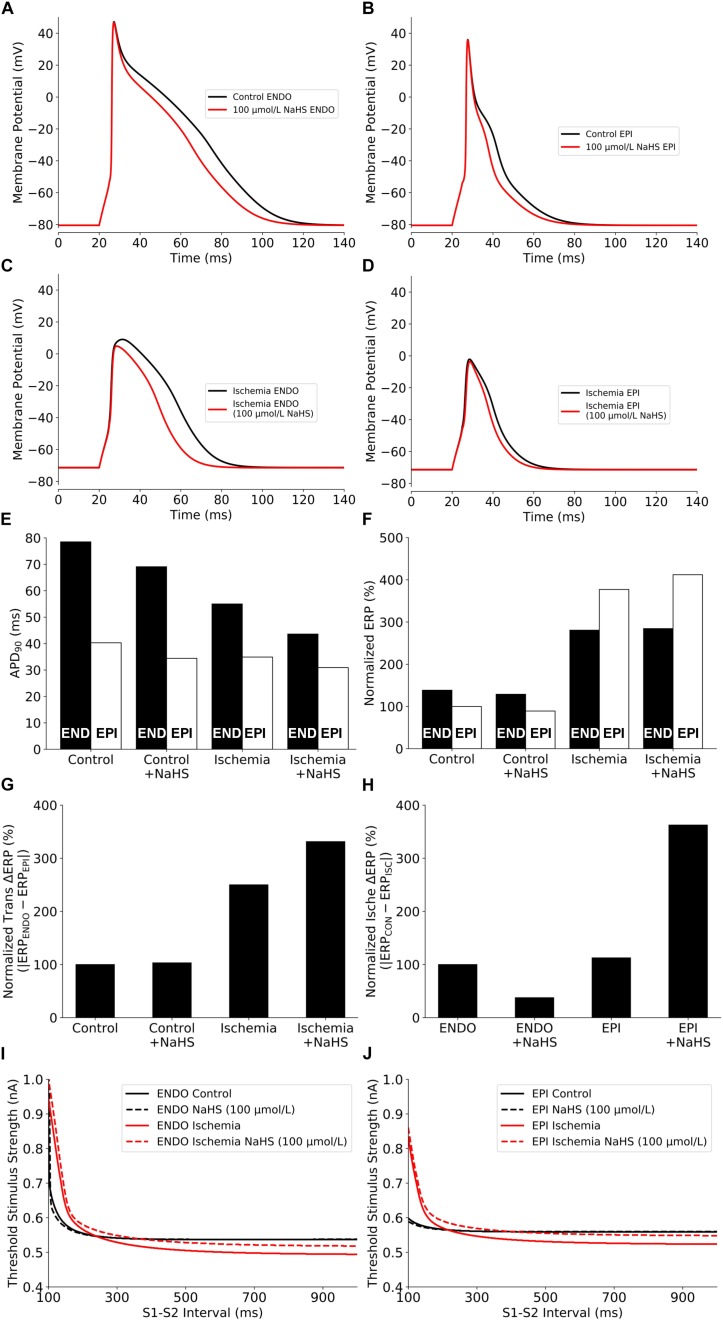
Summary of the effects of NaHS and ischemia on **(A–D)** AP morphology, **(E)** APD_90_, **(F)** Normalized ERP, **(G)** Normalized ERP transmural gradients between epicardial/endocardial cells, **(H)** Normalized ERP gradients between ischemic/non-ischemic cells, and **(I,J)** excitability. AP, action potential; APD, action potential duration; ERP, effective refractory period.

The effects of NaHS on the electrical APs of cardiac cells by integral actions of a reduced *I*_*CaL*_ and an enhanced *I*_*K,ATP*_ are presented in Figure 3. NaHS shortened the APD for both endocardial and epicardial cells (Figures 3A,B). Note that the results presented in Figures 3A,B are similar to those shown in Figure 1, where the *I*_*K,ATP*_ was blocked by a high concentration of the intracellular ATP, suggesting that the observed APD shortening by NaHS was mainly attributable to the reduced *I*_*CaL*_.

### Effects of NaHS in Ischemic Myocytes

NaHS may modulate differently the electrical activity of cardiac cells between ischemic and healthy conditions. To test this hypothesis, further simulations were conducted to evaluate the effect of NaHS on APs of ischemic cells, and results are shown in Figures 3C,D. Similar to the healthy condition, NaHS abbreviated APD for both endocardial and epicardial cells; however, it showed a greater APD abbreviation in endocardial than in epicardial cells. Such an observation reflects different electrophysiological responses of endocardial and epicardial cells to the same dose of NaHS. The use of 100 μmol/L NaHS barely affected the resting potential but decreased the AP amplitude (APA) of ischemic cells, which was more marked in the endocardial cell model.

### Effects of NaHS on Cellular Refractoriness and Excitability

The effects of NaHS on cellular APD and ERP, and the differences between the endocardial and epicardial cells are shown respectively in Figures 3E–G. NaHS abbreviated APD for both endocardial and epicardial cells in healthy and ischemic conditions; however, it had a different effect on ERP between ischemic and healthy conditions – NaHS decreased the ERP in healthy myocytes but increased it in ischemic myocytes. Such different effects of NaHS on modulating APD and ERP were attributed to the involvement of NaHS-*I*_*K,ATP*_, which suppressed the upstroke and amplitude of the AP, leading to a prolonged ERP in the ischemic condition. A non-uniformly modulated ERP between endocardial and epicardial cells produced an augmented ERP dispersion across the transmural ventricular wall, as illustrated in Figure 3G, with the measured transmural gradient of ERP increased by 3.1% and more significantly in the ischemic ventricular wall with ΔERP increased by 32.5%.

Further analyses were conducted to study the effect of NaHS on cellular excitability, measured reciprocally by the threshold of an external stimulus to evoke an AP. Results are shown in Figures 3I,J for the control and ischemic conditions with a low dose of NaHS at 100 μmol/L. In the healthy condition, NaHS did not affect the measured excitation threshold for either the endocardial or epicardial cell model but increased it in the ischemic condition, suggesting that NaHS reduced cellular excitability in the ischemic condition.

Note that the measured excitation threshold of the endocardial and epicardial cell models was affected by the ischemic condition, which is dependent on the S1–S2 intervals. In the ischemic condition, compared with the healthy condition, the measured threshold was lower at long intervals whereas greater at short intervals. Such a difference in the modulated cellular excitability by the ischemic condition was attributable to the combined effect of a prolonged ERP and a more depolarized resting potential as seen in the ischemic condition. Whereas the former increased the excitation threshold at short intervals, causing a rightward shift of the measured threshold curve (Figures 3I,J), the latter reduced the excitation threshold owing to a closer distance between the depolarized resting potential by ischemia and the threshold potential, which is easier to evoke an AP. However, if the resting potential was further elevated by more severe hyperkalemia and finally exceeded the threshold potential, it would always be harder to evoke an AP (i.e., a bigger excitation threshold) in ischemia than in healthy myocyte regardless of the S1–S2 interval.

### Temporal Vulnerability to Unidirectional Conduction Block

**FIGURE 4:**
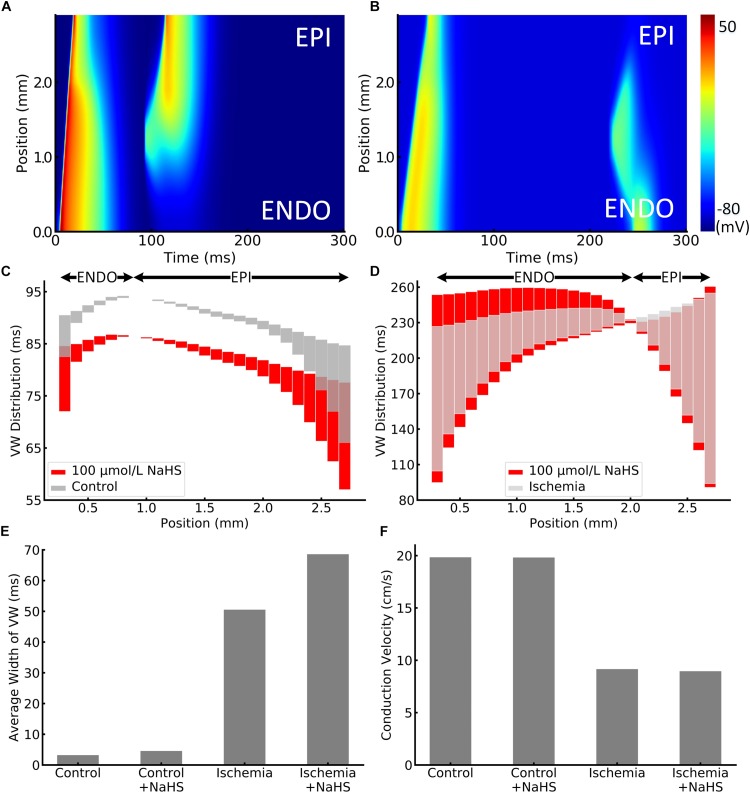
Illustration of unidirectional conduction block in **(A)** healthy and **(B)** ischemic 1D tissue strand. The vulnerable window (VW) distribution of different positions in strand is plotted in **(C)** healthy and **(D)** ischemic tissues. VWs were widened by NaHS in both healthy and ischemic tissues, presented as red bars. Arrows at the top represent the epicardial or endocardial direction toward which the impulse could be conducted. VWs that are less than 0.01 ms were ignored, as they might be incorrectly calculated because of the time precision in the model. The average width of VW in all cases is compared in panel **(E)**. In addition, the measured conduction velocity in 1D strand is shown in panel **(F)**.

The effect of NaHS on tissue’s vulnerability to genesis of unidirectional conduction block in response to a premature stimulus was also evaluated. Figures 4A,B presented examples of the simulated unidirectional conduction block in healthy and ischemic tissues. Figure 4A clearly shows that owing to the shorter APD in the epicardial region as compared with the endocardial portion, the S2 stimulus applied to the epicardial region (1.0–1.6 mm from the endocardial portion) produced an anterograde conduction. However, the same premature stimulus applied to the same location produced unidirectional conduction in the retrograde direction in ischemic tissues, owing to a reduced conduction velocity (CV) and the altered effective refractory properties. Unidirectional conduction block can only be induced at a certain time period (termed *vulnerable window* [VW]), or bidirectional block/propagation will happen. The computed overall distribution of VW across the tissue strand is shown in Figures 4C,D, where the light gray bars and red bars are the VW distribution with and without NaHS. The black arrows in these two panels indicated the direction of the unidirectional conduction evoked by the premature S2 stimulus, showing a change in the conduction direction. In healthy tissues, the direction of conduction changed at around 0.7 mm from the endocardial portion, and it happened in a more forward position around 1.9 mm in the ischemia case (Figure 4D). The average width of VW in four conditions is plotted in Figure 4E, in which it can be seen that ischemia greatly increased the width of the VW by nearly 16 times from 3.2 to 50.5 ms. Marked width of VW across the whole strand suggested a highly increased tissue susceptibility to unidirectional block in the ischemic condition.

When applying 100 μmol/L of NaHS, the width of VW was increased in both healthy and ischemic conditions, and the phenomenon was more significant in the ischemic tissues. Specifically, NaHS further increased the average width of VW from 50.5 to 68.6 ms in the ischemia condition, whereas it slightly increased the average VW width from 3.2 to 4.6 ms in healthy tissues (see Figure 4). The above observation suggested that NaHS could contribute to even higher risks in the ischemic tissues that are already fragile and susceptible to unidirectional block. The adverse effect of NaHS was observed in all locations throughout the strand notwithstanding the direction of conduction. Figure 4F presents the comparison of CV, where NaHS barely affected the CV in healthy tissues with only 0.1% decrement from 19.84 to 19.82 cm/s. In ischemic tissues, NaHS decreased the CV by 2.1% from 9.16 to 8.96 cm/s.

### Spatial Vulnerability of Tissue to Sustain Reentry

**FIGURE 5:**
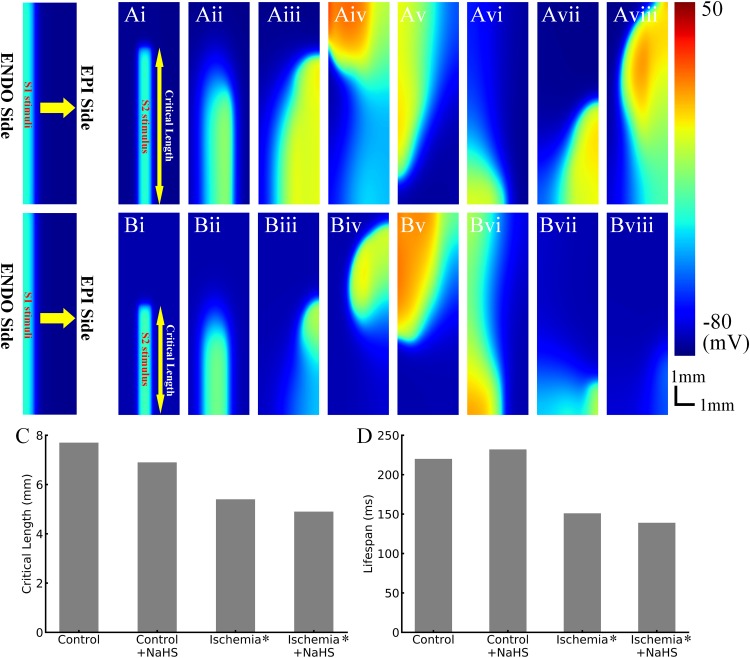
Representative snapshots of initiated spiral waves in **(Ai–Aviii)** healthy tissues and **(Bi–Bviii)** early ischemic tissues. **(C)** The critical length in control/health, control/health +100 μmol/L NaHS, early ischemia, and early ischemia +100 μmol/L NaHS. **(D)** The life span of initiated reentry in four cases.

The effects of NaHS on the dynamic behavior of reentrant excitation waves were also investigated. Premature stimulus during the VW in 2D tissues led to a unidirectional propagation of the impulse toward the anterograde direction, and this impulse further evolved into a spiral wave, as shown in Figure 5. After initiation of the spiral wave, it required a minimum tissue’s substrate to sustain, the *critical length* of which can be used as an index to quantify tissue susceptibility for maintaining reentry. In the healthy condition, the measured critical length was 7.7 mm (Figures 5Ai–Aviii), which decreased by almost 30% to 5.4 mm in the early phase of ischemic tissues (Figures 5Bi–Bviii). This was attributed to the reduced CV and APD in ischemic tissues, producing a short wavelength and thus facilitating the survival of the paired spiral waves.

NaHS measuring 100 μmol/L decreased the critical length in both healthy tissues and ischemic tissues (Figure 5C), which was also attributable to the shortening of APD and wavelength of excitation.

### Dynamics of Spiral Waves

Spiral waves in the 2D model of transmural ventricular tissues were unstable in both healthy and ischemic tissues, leading them to self-termination with a finite life span. As shown in Figure 5D, in the healthy tissues, the life span of spiral wave was about 220 ms, which was prolonged to 232 ms by NaHS. In the tissue model with early phase of ischemia, the life span of spiral wave was about 151 ms, which changed to about 139 ms in the NaHS condition.

## Discussion

Although the common industrial air pollutant H_2_S is hazardous to human cardiovascular system, its underlying mechanisms have not been elucidated yet. In this study, we computationally investigated the functional influence of H_2_S on cardiac electrical excitations in healthy and ischemic conditions on the basis of experimental data of NaHS (a donor of H_2_S). Our major findings are as follows: (i) at the cellular level, NaHS shortens APD, which was more significant in the ischemia condition, whereas it decreased ERP in healthy but increased it in the ischemic condition; (ii) NaHS augmented the ERP difference between epicardial and endocardial myocytes (i.e., ΔERP) in both healthy and ischemia conditions and augmented the ERP difference between ischemic and non-ischemic cells; (iii) at the tissue level, it increased tissue vulnerability for initiation and maintenance of reentry, which is more significant in the ischemic condition. It also prolonged the life span of reentry. All taken together, these findings provide explanations to the pro-arrhythmic effects of H_2_S, especially in the ischemic conditions.

### Mechanisms of Pro-arrhythmogenesis

#### Action Potential Duration and Effective Refractory Period Abbreviation

NaHS shortens APD in both healthy and ischemic myocytes through an integrated action of depressed *I*_*CaL*_ and enhanced *I*_*K,ATP*_. In simulation, APD abbreviation was more obvious in ischemia owing to more opened K-ATP channels. However, changes of ERP were different in these two situations, whereas NaHS decreased the ERP in healthy myocytes but increased it in ischemic myocytes, which is also attributable to the involvement of *I*_*K,ATP*_ in ischemia (but not in health) that enhanced the repolarization current and reduced the amplitude of APs. NaHS thus prolonged the ERP of ischemic myocytes through its action on increasing *I*_*K,ATP*_.

#### NaHS Augmented the Effective Refractory Period Dispersion

The transmural dispersion of ERP, measured by the ERP difference (i.e., ΔERP) between epicardial and endocardial myocytes, was augmented by NaHS in both healthy and ischemia conditions. In the healthy condition, NaHS augmented ΔERP as it decreased ERP more in the epicardial cells (by 9 ms) than in endocardial cells (by 8 ms) (see Figure 3F), resulting in a 1-ms increment in transmural ΔERP (Figure 3G). In the ischemia condition, NaHS also increased ΔERP but not in the same way as in healthy tissues. Instead, NaHS increased the ERP in both cell types as the *I*_*K,ATP*_ was involved in the ischemia condition. However, such ERP increasing effect was more significant in epicardial myocytes (up to 29 ms) than in endocardial myocytes (only 3 ms), as illustrated in the two data groups in the right side of Figure 3F. The unevenly altered ERP in epicardial and endocardial cells contributed to a great enhancement of transmural ΔERP and further increased it by 26 ms. In addition, NaHS also increased the ERP difference between ischemic and non-ischemic epicardial myocytes (Figure 3H). The observation suggested another potential pro-arrhythmic influence of NaHS when local ischemia happened, as the ERP dispersion between the local ischemic areas and the other healthy areas in the epicardium would possibly lead to arrhythmogenesis.

#### Increased Tissue Vulnerability for Arrhythmogenesis

The augmented repolarization dispersion by NaHS led to the increased temporal vulnerability of tissue to unidirectional conduction block in response to a premature stimulus (Figure 4). In addition, NaHS decreased the CV of excitation waves, which also contributed to the increased width of the measured VW that was exacerbated by the ischemic condition.

#### Decreased Minimal Substrate Size to Sustain Reentry

NaHS decreased the critical length in healthy and ischemic tissues for sustaining reentry, suggesting an important role in facilitating reentrant arrhythmia. This can be attributable to the abbreviated APD and ERP, resulting in a shorter excitation wavelength that reciprocally characterizes the spatial vulnerability of tissues (Rensma et al., 1988; Zhang et al., 2008).

#### Increased Life Span of Reentry

NaHS prolonged the life span of reentry in healthy tissues but not in ischemia. Owing to a limited tissue size (only 3 mm in transmural width) in this study, the initiated spiral wave always self-terminated with finite life span, which was determined by the interplay between the refractory distance of reentry and the transmural ERP gradient of the tissues. In one way, a short refractory distance (can be simply defined as the product of CV and ERP) of an excitation wave needs a small tissue size for accommodating the leading circle of reentry (Comtois et al., 2005), which helps to sustain the reentry. In the other way, the transmural gradient of ERP induces the tip of reentry to drift – a larger ERP gradient produces a more meandering of the tip to run out of the boundary, leading to self-termination. In our simulation, the spiral wave self-terminated in the control case owing to the intrinsic ventricular heterogeneity. NaHS decreased the refractory distance but did not increase the ERP gradient too much, and the two factors together resulted in a prolonged life span of reentry.

In the ischemic condition, the depressed excitability led to a prolonged ERP, and the refractory distance was enlarged despite a decreased CV. In addition, the augmented ERP dispersion also contributed to the termination of the spiral wave by accelerating the drifts. In this case, these factors interplayed, resulting in a shorter life span of spiral wave in ischemic tissues. NaHS further prolonged ERP by enhancing *I*_*K,ATP*_, and it also increased the ERP dispersion; these effects together terminated the spiral wave earlier.

### Potential Limitations of This Study

The limitations of the Pandit et al. (2001) model were discussed elsewhere (Terkildsen et al., 2008; Gattoni et al., 2016). In the simulations, the effects of H_2_S on cardiac ion channels were based on experimental data of a H_2_S donor, NaHS, as NaHS is superior to the direct administration of H_2_S for dose control, data reproducibility, and negligible effects on the cellular Na^+^ concentration and pH (Zhong et al., 2003, 2010).

In single-cell model development, data on the effects of NaHS on *I*_*CaL*_ and *I*_*K,ATP*_ were based on extant data from rats as much as possible to avoid potential species dependence (Furukawa et al., 1991). However, it should be of concern that the interspecies differences regarding the transmural *I*_*K,ATP*_ may exist. For example, experiments conducted on rabbit (Qi et al., 2000) or cat (Furukawa et al., 1991) showed a greater *I*_*K,ATP*_ activation in epicardial cells than in endocardial cells, but this is not applicable to rats according to the rat experiments (Shimokawa et al., 2007), which reported smaller half-maximal channel inhibition (IC_50_) of *I*_*K,ATP*_-ATP curve using either inside–out patch recordings or open cell-attached patch recordings, suggesting less *I*_*K,ATP*_ activation in epicardial cells than endocardial cells when facing the same decrement of intracellular ATP. Owing to the uncertainty of interspecies difference as well as the insufficient experimental data regarding transmural *I*_*K,ATP*_ difference in rats, the transmural heterogeneity of *I*_*K,ATP*_ was not incorporated in this study, and the conclusion should be carefully interpreted in clinical study considering the significant electrophysiological differences between human and rats. In 2D simulations, we only considered an earlier phase of ischemia as it was difficult to initiate the spiral wave in a tissue with conditions of 10-min ischemia. Specifically, the largely increased ERP led to a rather large leading circle that required large tissue size for spiral wave tip’s meandering, and the limited size of a rat’s transmural tissues will no longer be able to accommodate the spiral wave in the late phase of ischemia; thus, we did not conduct the simulation on tissues with conditions of 10-min ischemia.

Note that ischemia will also create the ERP dispersion between ischemic and non-ischemic areas, and NaHS could further enhance the dispersion in the epicardium. The role of such augmented ERP dispersion around the “border zone” of ischemic region may be pro-arrhythmic (Picard et al., 1999; Bernus et al., 2005; Trénor et al., 2007). In the presence of NaHS, how it increases the vulnerability of tissue to cardiac arrhythmias warrants future study.

## Conclusion

The pro-arrhythmic effects of H_2_S arise as a consequence of different alterations of electrical properties in epicardial and endocardial myocytes through modest changes in ion channel conductance, resulting in abbreviated ERP, augmented transmural ERP dispersion, and depressed cell excitability, leading to increased tissue susceptibility for initiation and maintenance of cardiac arrhythmia. The pro-arrhythmic effect of H_2_S was more significant in ischemic tissues, providing a novel insight into higher risks among cardiac ischemia population to H_2_S-induced ventricular arrhythmias.

## Materials and Methods

### Modeling K-ATP Channel in Epicardial and Endocardial Myocytes

We used a detailed mathematical model of K-ATP channel developed by Ferrero et al. (1996). More details of the model can be found in the Supplementary Material. Briefly, *I*_*K,ATP*_ is expressed as follows:

(1) $$I_{K,\mathrm{ATP}}={\text{g}}_{0}\,{\left(\frac{{\text{[K}}^{+}]o}{5.4}\right)}^{0.24}\,f_{\text{M}}\,f_{\text{N}}\,f_{\text{T}}\,f_{\text{ATP}}\,\left(V-E_{\text{K}}\right),$$

where g_0_ represents the maximum channel conductance (nS); [K^+^]_*o*_ is the extracellular concentration of K^+^ (mmol/L); factors *f*_*M*_, *f*_*N*_, *f*_*T*_ respectively denote the effects of intracellular Mg^2+^, Na^+^, and temperature. *V* and *E*_*K*_ represent the membrane potential and reverse potential of K^+^, respectively, (mV); and *f*_*ATP*_ is the fraction of opened channels. The model was further modified to incorporate species- and cell-type dependence and validated on available experimental data (see Supplementary Figure 1).

### Modeling Effects of Exogenous H_2_S on Ion Channels

In experiments, NaHS was used in most cases as H_2_S donor rather than the direct administration of H_2_S. Therefore, our simulations were based on experimental data of NaHS. Experimental data have shown that NaHS increased K-ATP, whereas it reduced the Ca-L channel current in rat cardiac cells. Therefore, the dose-dependent effect of NaHS on *I*_*K,ATP*_ and *I*_*CaL*_ was modeled by the following:

(2) $$I_{K,\mathrm{ATP}}^{\text{NaHS}}=\left(1+\frac{0.71}{1+{\left(K_{m,\mathrm{KATP}}/N\,a\,H\,S\right)}^{0.95}}\right)\,I_{K,\mathrm{ATP}}$$

(3) $$I_{\text{CaL}}^{\text{NaHS}}=\left(1-\frac{0.7}{1+{\left(K_{m,\mathrm{CaL}}/N\,a\,H\,S\right)}^{0.77}}\right)\,I_{\text{CaL}}$$

These equations were fitted to experimental data (Sun et al., 2008; Zhong et al., 2010; Zhang et al., 2012), resulting in *K*_*m,KATP*_ as 28.57 μmol/L and *K*_*m,CaL*_ as 376.66 μmol/L. Fitting results and experimental data are available in Supplementary Figure 2.

### Single-Cell Model

#### Healthy Rat Ventricular Myocyte Model

The above-described *I*_*K,ATP*_ was incorporated into the Pandit et al. (2001) model [with corrected equations by Terkildsen et al. (2008)] for the AP of rat ventricular myocytes. [ATP]_*i*_ and [ADP]_*i*_ were set to 6,800 and 15 μmol/L for healthy tissues (i.e., normoxic condition) based on measured nucleotide data as reported in a previous study (Weiss et al., 1992). APs were evoked in a same way as Pandit et al. (2001), that is, by a series of supra-threshold stimuli (S1; 1 Hz) with an amplitude of 0.6 nA and duration of 5 ms. Detailed methods on measuring APD, ERP, and excitability using the model are described in the Supplementary Material.

#### Ischemic Rat Ventricular Myocyte Model

For simulating the effect of ischemia on cellular APs, three major conditions were considered (i.e., hyperkalemia, anoxia, and acidosis), as considered in Shaw and Rudy’s study (Shaw and Rudy, 1997). Simulations of the effect of 10-min ischemia on nucleotide concentrations, ion concentrations, and channel current conductance are detailed as follows:

##### Hyperkalemia

(4) $${\text{[K}}^{+}]{}_{\text{o}}=8.0\mathrm{mmol}/L$$

The elevated extracellular K^+^ were based on rat experimental data as reported in Fiolet et al. (1984), Knopf et al. (1990) and Wilde et al. (1990).

##### Acidosis

(5) $${\text{g}}_{\mathrm{Na},\mathrm{acid}}=0.75\cdot {\text{g}}_{\text{Na}}$$

(6) $${\text{g}}_{\mathrm{CaL},\mathrm{acid}}=0.75\cdot {\text{g}}_{\text{CaL}}$$

Conductance reduction of these two channels was based on the observations in rat ventricular myocytes (Yatani and Goto, 1983; Yatani et al., 1984).

##### Anoxia

(7) $${\text{[ATP]}}_{\text{i}}=4,600\,\mu \,\mathrm{mol}/L$$

(8) $${\text{[ADP]}}_{\text{i}}=99\,\mu \,\mathrm{mol}/L$$

(9) $$P_{\mathrm{CaL},\mathrm{ATP}}=\frac{1}{1+{\left(\frac{K\,\text{m}}{{\text{[ATP]}}_{\text{i}}}\right)}^{H}}$$

Data of nucleotide levels in ischemia were obtained from Weiss et al. (1992). *P*_*CaL,ATP*_ represents the influence of anoxia on the open fraction of Ca-L channel; and *K*_*m*_ = 1,400 μmol/L and *H* = 2.6. The effects of the above modifications on AP configurations were validated for epicardial and endocardial myocytes using available experimental data. Detailed validation results are presented later in this section.

In addition to the 10-min ischemia model, an earlier phase of ischemia was also considered for a transient stage in 2D simulation. The parameters for the ischemic condition during the transient stage are as follows:

(10) $${\text{[K}}^{+}]{}_{\text{o}}=6.4\mathrm{mmol}/L$$

(11) $${\text{g}}_{\mathrm{Na},\mathrm{acid}}=0.85\cdot {\text{g}}_{\text{Na}}$$

(12) $${\text{g}}_{\mathrm{CaL},\mathrm{acid}}=0.85\cdot {\text{g}}_{\text{CaL}}$$

(13) $${\text{[ATP]}}_{\text{i}}=5500\,\mu \,\mathrm{mol}/L$$

(14) $${\text{[ADP]}}_{\text{i}}=64.6\,\mu \,\mathrm{mol}/L$$

The relationship between [ATP]_*i*_ and [ADP]_*i*_ was calculated by fitting the calculation function proposed by Bao et al. (2013).

#### Validation of Single-Cell Model for Control and Ischemic Conditions

**FIGURE 6:**
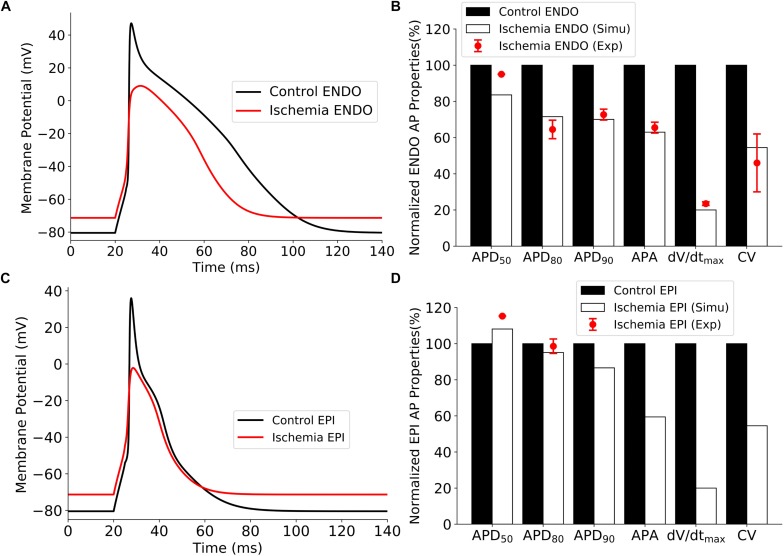
Effect of ischemia on ventricular APs. **(A)** APs of endocardial myocytes for control and 10 min after the onset of ischemia. **(B)** Comparison of simulation and experimental data of AP characteristics for control and ischemic conditions for endocardial myocytes. **(C)** APs of epicardial myocytes for control 10 min after the onset of ischemia. **(D)** Comparison of simulation and experimental data of AP characteristics for control and ischemic conditions for epicardial myocytes. AP, action potential.

Validations of the developed single-cell model of the rat ventricular APs are shown in Figure 6 for the control and ischemic conditions. The simulated ischemic condition as stated above based on the work of Shaw and Rudy (1997) abbreviated APs in both epicardial and endocardial myocytes; decreased their APA, maximum upstroke velocity (dV/dt_max_), and CV (measured in 1D tissue strand model); but elevated the resting potential from −80 to around −71 mV. These simulated effects of ischemic condition matched well to experimental observations from rat and other species (Bélichard et al., 1991; De Diego et al., 2008), validating the developed rat ventricle cell models. Validation results are illustrated in Figure 6. All the data sources are available in Supplementary Table 1.

### Transmural Ventricular Tissue Model

A multicellular tissue model for simulating the AP propagation was constructed using the well-established mono-domain equation (Clayton et al., 2011):

(15) $$\frac{\partial {\text{V}}_{\text{m}}}{\partial \text{t}}=\nabla \cdot \left(\text{D}\,\nabla {\text{V}}_{\text{m}}\right)-\frac{I_{\text{ion}}}{C_{\text{m}}}$$

where **D** is the diffusion coefficient matrix describing the intercellular electrical coupling via gap junctions. When considering the 1D tissue strand, Eq. 15 can be further organized as follows:

(16) $$\frac{\partial {\text{V}}_{\text{m}}}{\partial \text{t}}=D\,\left(\frac{\partial^{2}{\text{V}}_{\text{m}}}{\partial {\text{x}}^{2}}\right)-\frac{I_{\text{ion}}}{C_{\text{m}}}$$

where *D* is a scalar value that is set to 1.8 × 10^–2^ mm^2^/ms in the 1D tissue model, giving a transmural CV of 19.84 cm/s, within the measured range of 14.6 to 102 cm/s in rat ventricular tissues (Sedmera et al., 2016). As the ventricular wall in rat heart is rather thin, a length of 3 mm of transmural 1D strand was constructed with 2 mm designated as endocardial and 1 mm epicardial tissues, which was inconsistent with our previous study (Zhang et al., 2009). The spatial resolution dx was set to 0.1 mm, close to the length of single ventricular myocyte (80–150 μm); thus, the constructed strand contained 20 nodes for endocardial and 10 nodes for epicardial tissues.

The 2D transmural tissue model was constructed by expanding the 1D strand in the *y* direction, thus forming a 3 × 10 mm^2^ tissue sheet. The spatial resolution in the *y* direction dy is the same as dx. Anisotropic cell-to-cell electrical coupling CV was assumed in this study. Specifically, the fiber orientation was set to vertical, and the diffusion coefficients were set to 1.8 × 10^–2^ mm^2^/ms in the fiber orientation and 7.2 × 10^–2^ mm^2^/ms, which were perpendicular to the fiber orientation [i.e., coefficient ratio D_⊥_:D_| |_ = 1:4 according to Benson et al. (2008)]. The above settings gave a transmural CV of 19.84 cm/s and a longitudinal CV of 41.97 cm/s, approximately in a ratio of 1:2 in healthy tissues. In the ischemic condition, the coefficient was decreased by 20% to mimic the reduced intercellular coupling.

### Measurement of Vulnerability to Unidirectional Conduction Block – Temporal Vulnerability

Temporal vulnerability to reentrant arrhythmia was quantitatively measured as the width of VW in 1D tissue model. Standard S1–S2 protocol (Zhang et al., 2009) was used. Specifically, supra-threshold stimuli (S1) were applied to the 0.3-mm segment at the endocardial end for five times at 1 Hz. Then a premature stimulus S2 was applied to a 0.6-mm segment with a time delay Δt after S1. At a certain period that just after the refractory tail of S1 induced AP wavefront, S2 could produce a solitary wave that propagated in the anterograde but not retrograde direction, or vice versa. This was due to the different refractory durations between two directions. If S2 was applied too early, propagation failed in both directions and unidirectional conduction block could not be formed. On the other hand, if it was applied too late, bidirectional conduction could occur. The time period during which a S2 stimulus evoked unidirectional conduction block is referred to as VW.

Vulnerable window at each point in the 1D strand (except the 0.3-mm segments at the two ends of strand owing to the 0.6-mm stimulating length) was measured. In our simulation, the S1–S2 protocol was iteratively performed for every point until VWs across all strand were determined. The S1 stimuli were always applied to the first 0.3-mm endocardial segment in every measurement. As for the premature stimulus S2, the stimulating length was 0.6 mm, but its location was not fixed because every point on the strand had its own VW distribution that is to be determined in the simulation. For any given point, noted *x*, S2 was given to the segment from (*x*−0.3) mm to (*x*+0.3) mm after the S1 stimulus was applied. The interval between S2 and the last S1 (termed S1–S2 interval) was also not fixed, and different S1–S2 intervals would produce bidirectional conduction block (S2 too early), bidirectional conduction block (S2 too late), or unidirectional conduction block (S2 in VW). The time duration that S2 produced unidirectional conduction block was recorded as the width of VW for the point *x*.

### Measurement of Critical Size of Tissue to Support Reentry – Spatial Vulnerability

Using a similar S1–S2 protocol in 2D tissues may initiate the reentrant excitation wave. S1 stimuli were applied to the first 0.3 mm of cells in endocardial side (left side) for five times at 1 Hz. Then the premature stimulus S2 was applied during the VW to a local vertical area at the bottom of the tissues, with fixed width of 0.6 mm and variable length (or height) from 3.0 to 8.0 mm. The center of stimulated area was 1.3 mm away from the endocardial side (left side) and was consistent in all cases (i.e., control, NaHS, ischemia, and ischemia + NaHS) to avoid location-dependent variation.

During VW, excitation wave evoked by S2 stimulus could be unidirectional conduction, which evolves into spiral wave. However, the spiral wave could sustain only when a sufficient length of S2 was given, as the spiral wave required space to survive. Or if the length of S2 was too small, the tip of the spiral wave would hit the bottom boundary of the tissues and would self-terminate. Therefore, a certain length (termed *critical length*) could represent the spatial vulnerability of tissues to reentry.

In 2D simulations, an earlier phase of ischemia was also considered, with mild hyperkalemia from 5.4 to 6.4 mmol/L, 15% decrease of calcium and sodium channel conductance, and a reduction of intracellular ATP from 6,800 to 5,500 μmol/L. Equations in this condition are listed in *Ischemic Rat Ventricular Myocyte Model*, that is, Eqs 10–14.

## Data Availability Statement

All datasets generated for this study are included in the article/Supplementary Material.

## Author Contributions

HZ and ZW conceptualized the idea of the manuscript. HZ provided the basic mythological structure. SGZ, SZZ, and XF developed the relevant codes in the single-cell model. SGZ, XF, and WW developed the relevant codes in 1D and 2D tissue models. HZ, SZZ, ZL, and DJ analyzed the simulation results, including the validation of cell model output and the spiral wave results. SGZ drafted the original manuscript. SGZ, HZ, and ZW wrote the manuscript.

## Conflict of Interest

The authors declare that the research was conducted in the absence of any commercial or financial relationships that could be construed as a potential conflict of interest.

## References

[B1] Abramochkin D. V. Moiseenko L. S. Kuzmin V. S. (2009). The effect of hydrogen sulfide on electrical activity of rat atrial myocardium. *Bull. Exp. Biol. Med.* 147 683–686. 10.1007/s10517-009-0607-y 19902056

[B2] Bao L. Taskin E. Foster M. Ray B. Rosario R. Ananthakrishnan R. (2013). Alterations in ventricular KATP channel properties during aging. *Aging Cell* 12 167–176. 10.1111/acel.12033 23173756 PMC3551995

[B3] Bates M. N. Garrett N. Shoemack P. (2002). Investigation of health effects of hydrogen sulfide from a geothermal source. *Arch. Environ. Health* 57 405–411. 10.1080/00039890209601428 12641180

[B4] Bélichard P. Pruneau D. Rouet R. Salzmann J. L. (1991). Electrophysiological responses of hypertrophied rat myocardium to combined hypoxia, hyperkalemia, and acidosis. *J. Cardiovasc. Pharmacol.* 17 S141–S145. 1715465 10.1097/00005344-199117002-00034

[B5] Benson A. P. Aslanidi O. V. Zhang H. Holden A. V. (2008). The canine virtual ventricular wall: a platform for dissecting pharmacological effects on propagation and arrhythmogenesis. *Prog. Biophys. Mol. Biol.* 96 187–208. 10.1016/j.pbiomolbio.2007.08.002 17915298

[B6] Bernus O. Zemlin C. W. Zaritsky R. M. Mironov S. F. Pertsov A. M. (2005). Alternating conduction in the ischaemic border zone as precursor of reentrant arrhythmias: a simulation study. *Europace* 7 S93–S104. 16102507 10.1016/j.eupc.2005.03.018

[B7] Bian J.-S. Yong Q. C. Pan T.-T. Feng Z.-N. Ali M. Y. Zhou S. (2006). Role of hydrogen sulfide in the cardioprotection caused by ischemic preconditioning in the rat heart and cardiac myocytes. *J. Pharmacol. Exp. Ther.* 316 670–678. 10.1124/jpet.105.092023 16204473

[B8] Chan C.-S. Lin Y.-K. Kao Y.-H. Chen Y.-C. Chen S.-A. Chen Y.-J. (2018). Hydrogen sulphide increases pulmonary veins and atrial arrhythmogenesis with activation of protein kinase C. *J. Cell Mol. Med.* 22 3503–3513. 10.1111/jcmm.13627 29659148 PMC6010708

[B9] Clayton R. H. Bernus O. Cherry E. M. Dierckx H. Fenton F. H. Mirabella L. (2011). Models of cardiac tissue electrophysiology: progress, challenges and open questions. *Prog. Biophys. Mol. Biol.* 104 22–48. 10.1016/j.pbiomolbio.2010.05.008 20553746

[B10] Comtois P. Kneller J. Nattel S. (2005). Of circles and spirals: bridging the gap between the leading circle and spiral wave concepts of cardiac reentry. *Europace* 7 S10–S20. 16102499 10.1016/j.eupc.2005.05.011

[B11] De Diego C. Pai R. K. Chen F. Xie L.-H. De Leeuw J. Weiss J. N. (2008). Electrophysiological consequences of acute regional ischemia/reperfusion in neonatal rat ventricular myocyte monolayers. *Circulation* 118 2330–2337. 10.1161/CIRCULATIONAHA.108.789149 19015404 PMC2730415

[B12] Ferrero J. M. Sáiz J. Thakor N. V. (1996). Simulation of action potentials from metabolically impaired cardiac myocytes: role of ATP-sensitive K^+^ current. *Circ. Res.* 79 208–221. 10.1161/01.res.79.2.208 8755997

[B13] Finnbjornsdottir R. G. Carlsen H. K. Thorsteinsson T. Oudin A. Lund S. H. Gislason T. (2016). Association between daily hydrogen sulfide exposure and incidence of emergency hospital visits: a population-based study. *PLoS One* 11:e0154946. 10.1371/journal.pone.0154946 27218467 PMC4878737

[B14] Finnbjornsdottir R. G. Oudin A. Elvarsson B. T. Gislason T. Rafnsson V. (2015). Hydrogen sulfide and traffic-related air pollutants in association with increased mortality: a case-crossover study in Reykjavik, Iceland. *BMJ Open* 5:e007272. 10.1136/bmjopen-2014-007272 25854971 PMC4390682

[B15] Fiolet J. W. T. Baartscheer A. Schumacher C. A. Coronel R. Ter Welle H. F. (1984). The change of the free energy of ATP hydrolysis during global ischemia and anoxia in the rat heart: Its possible role in the regulation of transsarcolemmal sodium and potassium gradients. *J. Mol. Cell. Cardiol.* 16 1023–1036. 10.1016/s0022-2828(84)80015-2 6520874

[B16] Fiordelisi A. Piscitelli P. Trimarco B. Coscioni E. Iaccarino G. Sorriento D. (2017). The mechanisms of air pollution and particulate matter in cardiovascular diseases. *Heart Fail. Rev.* 22 337–347. 10.1007/s10741-017-9606-7 28303426

[B17] Furukawa T. Kimura S. Furukawa N. Bassett A. L. Myerburg R. J. (1991). Role of cardiac ATP-regulated potassium channels in differential responses of endocardial and epicardial cells to ischemia. *Circ. Res.* 68 1693–1702. 10.1161/01.res.68.6.1693 2036719

[B18] Gattoni S. Røe ÅT. Frisk M. Louch W. E. Niederer S. A. Smith N. P. (2016). The calcium–frequency response in the rat ventricular myocyte: an experimental and modelling study. *J. Physiol.* 594 4193–4224. 10.1113/JP272011 26916026 PMC4967761

[B19] Godleski J. J. Verrier R. L. Koutrakis P. Catalano P. Coull B. Reinisch U. (2000). Mechanisms of morbidity and mortality from exposure to ambient air particles. *Res. Rep. Health Eff. Inst.* 91 5–88. 10817681

[B20] Johansen D. Ytrehus K. Baxter G. F. (2006). Exogenous hydrogen sulfide (H2S) protects against regional myocardial ischemia–reperfusion injury. *Basic Res. Cardiol.* 101 53–60. 10.1007/s00395-005-0569-9 16328106

[B21] Knopf H. Theising R. Moon C. H. Hirche H. J. (1990). Continuous determination of extracellular space and changes of K^+^, Na^+^, Ca2^+^, and H^+^ during global ischaemia in isolated rat hearts. *J Mol Cell Cardiol* 22 1259–1272. 10.1016/0022-2828(90)90062-7 2283684

[B22] Link M. S. Dockery D. W. (2010). Air pollution and the triggering of cardiac arrhythmias. *Curr. Opin. Cardiol.* 25 16–22. 10.1097/HCO.0b013e32833358cd 19881339 PMC3750956

[B23] Martinelli N. Olivieri O. Girelli D. (2013). Air particulate matter and cardiovascular disease: a narrative review. *Eur. J. Intern. Med.* 24 295–302. 10.1016/j.ejim.2013.04.001 23647842

[B24] Mills N. L. Donaldson K. Hadoke P. W. Boon N. A. MacNee W. Cassee F. R. (2009). Adverse cardiovascular effects of air pollution. *Nat. Clin. Pract. Cardiovasc. Med.* 6 36–44. 10.1038/ncpcardio1399 19029991

[B25] Pandit S. V. Clark R. B. Giles W. R. Demir S. S. (2001). A mathematical model of action potential heterogeneity in adult rat left ventricular myocytes. *Biophys. J.* 81 3029–3051. 10.1016/s0006-3495(01)75943-7 11720973 PMC1301767

[B26] Picard S. Rouet R. Ducouret P. Puddu P. E. Flais F. Criniti A. (1999). KATP channels and ‘border zone’arrhythmias: role of the repolarization dispersion between normal and ischaemic ventricular regions. *Br. J. Pharmacol.* 127 1687–1695. 10.1038/sj.bjp.0702704 10455327 PMC1566150

[B27] Qi X. Y. Shi W. B. Wang H. H. Zhang Z. X. Xu Y. Q. (2000). A study on the electrophysiological heterogeneity of rabbit ventricular myocytes the effect of ischemia on action potentials and potassium currents. *Sheng Li Xue Bao* 52 360–364. 11941387

[B28] Rensma P. L. Allessie M. A. Lammers W. J. Bonke F. I. Schalij M. J. (1988). Length of excitation wave and susceptibility to reentrant atrial arrhythmias in normal conscious dogs. *Circ. Res.* 62 395–410. 10.1161/01.res.62.2.395 3338122

[B29] Schlesinger R. B. (2007). The health impact of common inorganic components of fine particulate matter (PM2. 5) in ambient air: a critical review. *Inhal. Toxicol.* 19 811–832. 10.1080/08958370701402382 17687714

[B30] Sedmera D. Neckar J. Benes J. Jr. Pospisilova J. Petrak J. Sedlacek K. (2016). Changes in myocardial composition and conduction properties in rat heart failure model induced by chronic volume overload. *Front. Physiol.* 7:367. 10.3389/fphys.2016.00367 27610087 PMC4997968

[B31] Shaw R. M. Rudy Y. (1997). Electrophysiologic effects of acute myocardial ischemia: a theoretical study of altered cell excitability and action potential duration. *Cardiovasc. Res.* 35 256–272. 10.1016/s0008-6363(97)00093-x 9349389

[B32] Shimokawa J. Yokoshiki H. Tsutsui H. (2007). Impaired activation of ATP-sensitive K^+^ channels in endocardial myocytes from left ventricular hypertrophy. *Am. J. Physiol. Circ. Physiol.* 293 H3643–H3649. 17921319 10.1152/ajpheart.01357.2006

[B33] Sun Y.-G. Cao Y.-X. Wang W.-W. Ma S.-F. Yao T. Zhu Y.-C. (2008). Hydrogen sulphide is an inhibitor of L-type calcium channels and mechanical contraction in rat cardiomyocytes. *Cardiovasc. Res.* 79 632–641. 10.1093/cvr/cvn140 18524810

[B34] Terkildsen J. R. Niederer S. Crampin E. J. Hunter P. Smith N. P. (2008). Using Physiome standards to couple cellular functions for rat cardiac excitation–contraction. *Exp. Physiol.* 93 919–929. 10.1113/expphysiol.2007.041871 18344258

[B35] Trénor B. Romero L. Ferrero J. M. Sáiz J. Moltó G. Alonso J. M. (2007). Vulnerability to reentry in a regionally ischemic tissue: a simulation study. *Ann. Biomed. Eng.* 35 1756–1770. 10.1007/s10439-007-9353-3 17616818

[B36] Weiss J. N. Venkatesh N. Lamp S. T. (1992). ATP-sensitive K^+^ channels and cellular K^+^ loss in hypoxic and ischaemic mammalian ventricle. *J. Physiol.* 447 649–673. 10.1113/jphysiol.1992.sp019022 1593462 PMC1176056

[B37] Wilde A. A. M. Escande D. Schumacher C. A. Thuringer D. Mestre M. Fiolet J. W. T. (1990). Potassium accumulation in the globally ischemic mammalian heart. A role for the ATP-sensitive potassium channel. *Circ. Res.* 67 835–843. 10.1161/01.res.67.4.835 2119912

[B38] Yatani A. Brown A. M. Akaike N. (1984). Effect of extracellular pH on sodium current in isolated, single rat ventricular cells. *J. Membr. Biol.* 78 163–168. 10.1007/bf01869203 6325701

[B39] Yatani A. Goto M. (1983). The effect of extracellular low pH on the plateau current in isolated, single rat ventricular cells-A voltage clamp study. *Jpn. J. Physiol.* 33 403–415. 10.2170/jjphysiol.33.403 6314007

[B40] Zhang H. Kharche S. Holden A. V. Hancox J. C. (2008). Repolarisation and vulnerability to re-entry in the human heart with short QT syndrome arising from KCNQ1 mutation—A simulation study. *Prog. Biophys. Mol. Biol.* 96 112–131. 10.1016/j.pbiomolbio.2007.07.020 17905416

[B41] Zhang H. Tao T. Kharche S. Harrison S. M. (2009). Modelling changes in transmural propagation and susceptibility to arrhythmia induced by volatile anaesthetics in ventricular tissue. *J. Theor. Biol.* 257 279–291. 10.1016/j.jtbi.2008.12.004 19135456

[B42] Zhang R. Sun Y. Tsai H. Tang C. Jin H. Du J. (2012). Hydrogen sulfide inhibits L-type calcium currents depending upon the protein sulfhydryl state in rat cardiomyocytes. *PLoS One* 7:e37073. 10.1371/journal.pone.0037073 22590646 PMC3349658

[B43] Zhang Z. Huang H. Liu P. Tang C. Wang J. (2007). Hydrogen sulfide contributes to cardioprotection during ischemia–reperfusion injury by opening KATP channels. *Can. J. Physiol. Pharmacol.* 85 1248–1253. 10.1139/y07-120 18066126

[B44] Zhong G. Chen F. Cheng Y. Tang C. Du J. (2003). The role of hydrogen sulfide generation in the pathogenesis of hypertension in rats induced by inhibition of nitric oxide synthase. *J. Hypertens* 21 1879–1885. 10.1097/00004872-200310000-00015 14508194

[B45] Zhong G.-Z. Li Y.-B. Liu X.-L. Guo L.-S. Chen M. Yang X.-C. (2010). Hydrogen sulfide opens the KATP channel on rat atrial and ventricular myocytes. *Cardiology* 115 120–126. 10.1159/000260073 19940474

