# Supplementary material for: Pro-arrhythmic Effects of Hydrogen Sulfide in Healthy and Ischemic Cardiac Tissues: Insight From a Simulation Study
Source: Front Physiol. 2019 Dec 13;10:1482. doi: 10.3389/fphys.2019.01482 (PMC6923703; doi:10.3389/fphys.2019.01482)
Supplement: Supplementary file 1 [file Data_Sheet_1.pdf]

Supplementary Material

1 Modelling K-ATP Channel in Epicardial and Endocardial Myocytes

Ferrero et al. [1] developed a detailed mathematical model of K-ATP channel current and the dependence of the current density on ion concentrations ($[K^+]_o$, $[Mg^{2+}]_i$, and $[Na^+]_i$) and intracellular nucleotide levels ($[ATP]_i$ and $[ADP]_i$), which takes the form:

$$I_{K,ATP} = g_0 \left(\frac{[K^+]_o}{5.4}\right)^{0.24} f_M f_N f_T f_{ATP} (V - E_K) \quad (S1)$$

where g_0 represents the maximum channel conductance in the absence of Na^+, Mg^{2+}, and ATP. $[K^+]_o$ is the extracellular concentration of K^+ in mmol/L. Factors f_M, f_N, f_T are respectively denoting the effects of intracellular Mg^{2+}, Na^+, and temperature. V and E_K represent the membrane potential and reverse potential of K^+ respectively and f_{ATP} is the fraction of opened channels, which takes the form:

$$f_{ATP} = \frac{1}{1+([ATP]_i/K_m)^H} \quad (S2)$$

where $[ATP]_i$ is the intracellular concentration of ATP (μmol/L), K_m (μmol/L) represents the certain concentration of ATP that corresponds to 50% opening fraction of the K-ATP channels. H characterizes the slope of the curve.

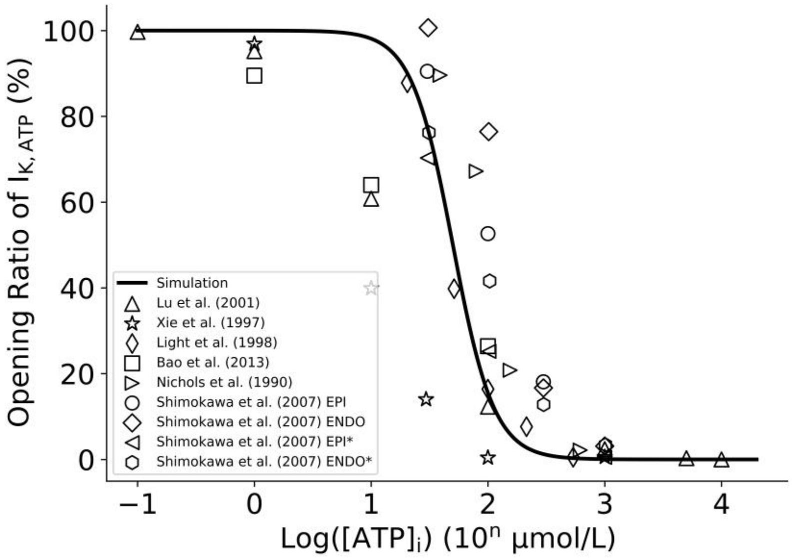

Supplementary Figure 1. Simulated ATP concentration dependent opening ration of K-ATP channel for EPI and ENDO cells, which were compared to experimental data. $[ADP]_i$ was fixed at 15 μmol/L as in [2].
* Two groups of experimental data in [3] were marked in Supplementary Figure 1, which are

separately the I_{KATP}-ATP relationship obtained from inside-out patch recordings (the group without asterisk), and that obtained from open cell-attached patch recordings (the group with asterisk).

Heidenreich et al. [2] modified the equations of K_m and H by considering two parameters α and β, which are related to species- and cell-type-dependence. The K_m and H are calculated as:

$$K_m = \alpha(35.8 + 17.9[\mathrm{ADP}]_\mathrm{i}^{0.256}) \quad \text{(S3)}$$

$$H = 1.3 + 0.74\beta\exp(-0.09[\mathrm{ADP}]_\mathrm{i}) \quad \text{(S4)}$$

These mathematical equations for $I_{K,ATP}$ were to be incorporated into the Pandit's model. However, as the equations were based on data from guinea pigs or other mammalian species, their parameters were refitted based on data from rat cardiomyocytes [4], resulting in the maximum channel conductance g_0 being 1150 nS.

Rat-dependent $I_{K,ATP}$ for ventricular myocytes was simulated based on experimental data from [3–8], which were used for adjusting α, β. The resultant fitting curve of f_{ATP} against $[ATP]_i$ based on the experimental data are shown in Supplementary Figure 1. Due to the insufficient experimental data, the transmural heterogeneity was not considered in this study. Based on the data fitting, we obtained parameters of α = 0.7 and β = 6 in both cell types.

2 Modelling effects of exogeneous H_2S on ion channels

Fitting results of the dose-dependent effect of NaHS on $I_{K,ATP}$ and I_{CaL} that modelled by Equations (2)(3) (in the paper) are shown in Supplementary Figure 2.

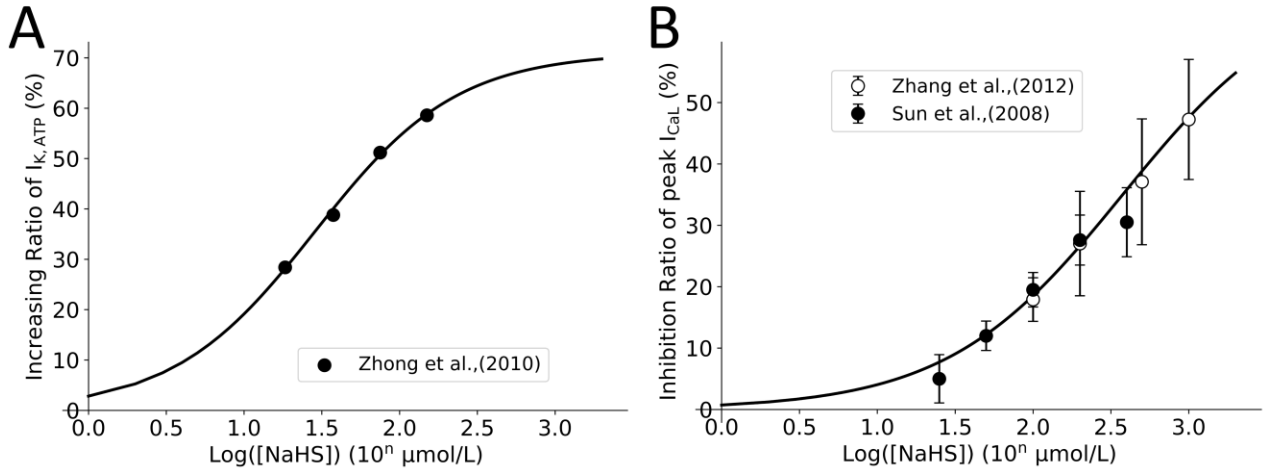

Supplementary Figure 2. Effects of NaHS on (A) enhancing $I_{K,ATP}$, data from Zhong et al. [9]; (B) inhibiting I_{CaL}, data from Zhang et al., Sun et al. [10,11].

3 Validation of single cell model for control and ischemic conditions

Supporting data for the Figure 6 (in the paper) are summarized in Supplementary Table 1, along with the literature references.

Supplementary Table 1. Summary of electrophysiological changes during early phase ischemia in ventricular myocytes of rats

	Ischemia Simulation		Ischemia Experiments		
Cell type	ENDO	EPI	ENDO (10min) [12]	ENDO (7min) [13]*	EPI (10 min) [14,15]
APD_{50} (%)	83.6%	108.1%	95.0%	-	115.4%
APD_{80} (%)	71.5%	95.1%†	-	64.5±5.1%	102.6%/94.6% †
APD_{90} (%)	70.1%	86.6%	72.7%	-	-
APA (%)	63.0%	59.8%	65.5±3.0%	60.9±4.6%	-
V_{max} (%)	20.0%	17.8%	23.5%	-	-
CV (%)	47.4%	41.4%	-	46.0±16.0%	-

* Action potential could not be elicited at 10 minutes in experiments, thus the record at 7 minutes is listed here.

† Note that the discrepancy between simulation and experimental results, or even between the experiments themselves mainly attributable to the relatively small duration value. In fact, the absolute value is rather close, e.g. APD_{90} decreased only 2ms, from 37 ms to 35 ms in Workman's study [14].

4 APD, ERP, and excitability measurements

Action potentials (AP) were evoked in a same way as Pandit et al. [16], i.e., by a series of supra-threshold stimuli (S1; 1 Hz) with an amplitude of 0.6 nA and duration of 5 ms. APD_{20}, APD_{50}, APD_{80}, APD_{90} were measured in the last AP as the time duration from the upstroke to the time at which the AP first went below 20%, 50%, 80%, 90% of the total amplitude.

The excitability was measured using S1-S2 protocol. In detail, suprathreshold stimuli S1 were applied, then a premature stimulus S2 with same stimulating duration as S1 (5 ms), but variable strength and time intervals was given after running S1 protocol for 5 seconds. For each time interval, the minimum stimulus strength of S2 that evokes an AP was measured. The minimum strength, or *threshold stimulus strength*, was plotted against S1-S2 interval, forming a L-shape curve. Generally, myocytes are less excitable if the curve is upper or right-shifted.

The ERP was measured using a similar S1-S2 protocol but in a 3 mm homogeneous 1D strand rather than in single cell, as used previously in [17]. Specifically, S1 stimuli were applied on the first three nodes (0.3 mm) of the strand, then a premature stimulus (S2) with same amplitude and duration as S1, but in variable time intervals, was also given to the first three nodes after running S1 protocol for 5 seconds. The S2 was iteratively applied by decreasing the time interval between S1 and S2 until the

S2 just failed to generate a successful excitation propagation, i.e., the action potential of the last cell on the strand was not evoked. . The smallest S1-S2 interval that S2-evoked impulse could propagate to the last cell was measured as ERP. The strand was homogenous with only one type of cells, for example, ERP of ischemic epicardial myocyte was measured on a 1D strand consisting of 30 ischemic epicardial cells. The ERP that obtained in 1D strand was more precise than that measured in single cell as it is hard to determine the threshold of S2-evoked AP amplitude that just enough to propagate, especially in ischemia conditions.

5 Sensitivity analysis

Sensitivity analyses regarding relationships of ΔAPD_{90}~$\Delta I_{K,ATP}$ and ΔAPD_{90}~ΔI_{CaL} were conducted considering ionic current variation range in experiments. The results are plotted in Supplementary Figure 3. It can be observed that, $I_{K,ATP}$ barely affected APD in either healthy epi or healthy endo cells. $I_{K,ATP}$ could affect APD only in ischemic condition (red lines) with a negative correlation, and the APD abbreviation effects were more significant in ischemic endo cells. As for the I_{CaL}, positive correlation can be found between APD and I_{CaL}.

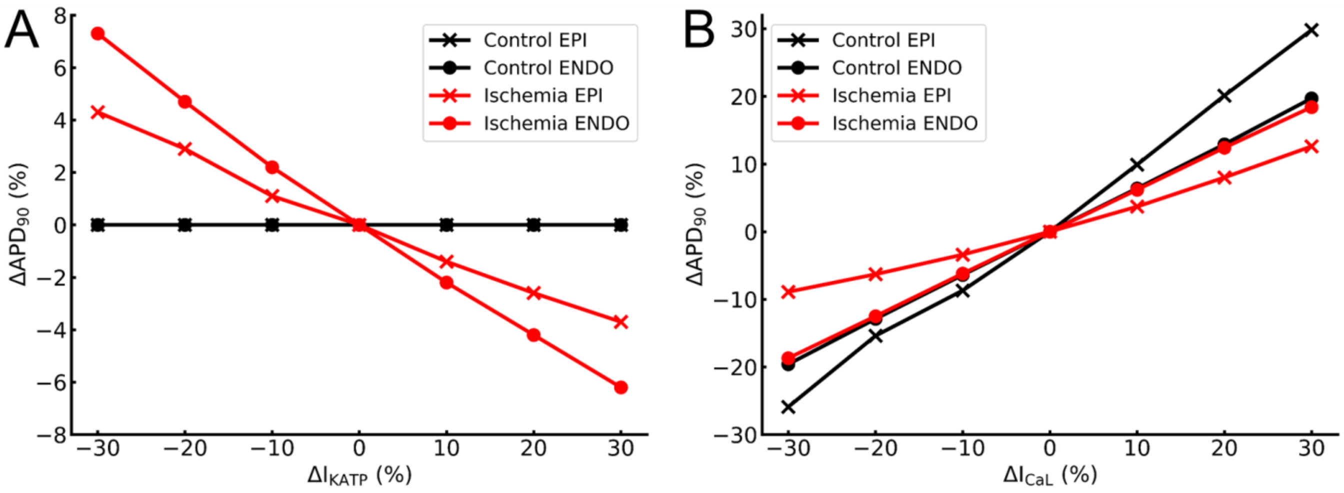

6 Supplementary Figure 3. Sensitivity analysis of APD variation regarding (A) I_{KATP} (B) I_{CaL}. An experimental variation range of these two currents from ±10% to ±30% were considered. References

[1] Ferrero JM, Sáiz J, Thakor N V. Simulation of action potentials from metabolically impaired cardiac myocytes: role of ATP-sensitive K+ current. Circ Res 1996;79:208–21.

[2] Heidenreich EA, Ferrero JM, Rodríguez JF. Modeling the human heart under acute ischemia. Patient-Specific Comput. Model., Springer; 2012, p. 81–103.

[3] Shimokawa J, Yokoshiki H, Tsutsui H. Impaired activation of ATP-sensitive K+ channels in endocardial myocytes from left ventricular hypertrophy. Am J Physiol Circ Physiol 2007;293:H3643--H3649.

[4] Nichols CG, Lederer WJ. The regulation of ATP-sensitive K+ channel activity in intact and permeabilized rat ventricular myocytes. J Physiol 1990;423:91–110.

[5] Lu T, Hoshi T, Weintraub NL, Spector AA, Lee H-C. Activation of ATP-sensitive K+ channels by epoxyeicosatrienoic acids in rat cardiac ventricular myocytes. J Physiol 2001;537:811–27.

[6] Xie LH, Takano M, Noma A. Development of inwardly rectifying K+ channel family in rat ventricular myocytes. Am J Physiol Circ Physiol 1997;272:H1741--H1750.

[7] Light P, Shimoni Y, Harbison S, Giles W, French RJ. Hypothyroidism decreases the ATP sensitivity of K ATP channels from rat heart. J Membr Biol 1998;162:217–23.

[8] Bao L, Taskin E, Foster M, Ray B, Rosario R, Ananthakrishnan R, et al. Alterations in ventricular KATP channel properties during aging. Aging Cell 2013;12:167–76.

[9] Zhong G-Z, Li Y-B, Liu X-L, Guo L-S, Chen M, Yang X-C. Hydrogen sulfide opens the KATP channel on rat atrial and ventricular myocytes. Cardiology 2010;115:120–6.

[10] Sun Y-G, Cao Y-X, Wang W-W, Ma S-F, Yao T, Zhu Y-C. Hydrogen sulphide is an inhibitor of L-type calcium channels and mechanical contraction in rat cardiomyocytes. Cardiovasc Res 2008;79:632–41.

[11] Zhang R, Sun Y, Tsai H, Tang C, Jin H, Du J. Hydrogen sulfide inhibits L-type calcium currents depending upon the protein sulfhydryl state in rat cardiomyocytes. PLoS One 2012;7:e37073.

[12] Bélichard P, Pruneau D, Rouet R, Salzmann JL. Electrophysiological responses of hypertrophied rat myocardium to combined hypoxia, hyperkalemia, and acidosis. J Cardiovasc Pharmacol 1991;17:S141--5.

[13] De Diego C, Pai RK, Chen F, Xie L-H, De Leeuw J, Weiss JN, et al. Electrophysiological consequences of acute regional ischemia/reperfusion in neonatal rat ventricular myocyte monolayers. Circulation 2008;118:2330–7.

[14] Workman AJ, MacKenzie I, Northover BJ. Do K ATP channels open as a prominent and early feature during ischaemia in the Langendorff-perfused rat heart? Basic Res Cardiol 2000;95:250–60.

[15] Workman AJ, MacKenzie I, Northover BJ. A KATP channel opener inhibited myocardial reperfusion action potential shortening and arrhythmias. Eur J Pharmacol 2001;419:73–83.

[16] Pandit S V, Clark RB, Giles WR, Demir SS. A mathematical model of action potential heterogeneity in adult rat left ventricular myocytes. Biophys J 2001;81:3029–51.

[17] Qu Z, Garfinkel A, Weiss JN. Vulnerable window for conduction block in a one-dimensional cable of cardiac cells, 1: single extrasystoles. Biophys J 2006;91:793–804.
